# Screening for Sexual Orientation in Psychiatric Emergency Departments

**DOI:** 10.5811/westjem.2014.12.22254

**Published:** 2015-01-06

**Authors:** Glenn W. Currier, Gregory Brown, Patrick G. Walsh, Shari Jager-Hyman, Sadia Chaudhury, Barbara Stanley

**Affiliations:** * University of Rochester Medical Center, Department of Psychiatry, Rochester, New York; † University of Rochester Medical Center, Department of Emergency Medicine, Rochester, New York; ‡ University of Pennsylvania School of Medicine, Department of Psychiatry, Philadelphia, Pennsylvania; § Columbia University School of Medicine, Department of Psychiatry, New York, New York

## Abstract

**Introduction:**

Our goal was to explore whether emergency department (ED) patients would disclose their sexual orientation in a research evaluation and to examine demographic and clinical characteristics of patients by self-identified sexual orientation.

**Methods:**

Participants (n=177) presented for psychiatric treatment at three urban EDs in New York City, Rochester, NY, and Philadelphia, PA. Participants were interviewed in the context of a larger study of a standardized suicide risk assessment. We assessed participants’ willingness to answer questions regarding sexual orientation along three dimensions: a self-description of sexual orientation, a self-description of sexual attraction, and the gender of any prior sexual partners.

**Results:**

No participants (0/177) refused to respond to the categorical question about sexual orientation, 168/177 (94.9%) agreed to provide information about prior sexual partners, and 100/109 (91.7%) provided information about current sexual attraction toward either gender. Of all 177 participants, 154 (87.0%) self-identified as heterosexual, 11 (6.2%) as bisexual, 10 (5.6%) as gay or lesbian, and 2 (1.1%) indicated they were not sure. As compared with heterosexual patients, lesbian, gay and bisexual (LGB) patients were significantly younger and more likely to be non-white, but did not differ significantly in terms of education, income, employment, or religious affiliation or participation. Further, LGB participants did not differ from self-identified heterosexual participants for lifetime suicide attempt rate or lifetime history of any mood, substance-related, psychotic spectrum, or other Diagnostic and Statistical Manual of Mental Disorders, 4th edition (DSM-IV) Axis I disorder. Of self-identified heterosexual participants 5.6% (5/89) reported sexual attraction as other than ‘only opposite sex,’ and 10.3% (15/142) of sexually active ‘heterosexual’ participants reported previous same-gender sexual partners.

**Conclusion:**

Assessing patients’ sexual orientation in the ED by a three-question approach appeared feasible in the ED and acceptable to ED patients. However, since many patients have sexual experiences not suggested by simple labels, self-report of sexual identity alone may not inform clinicians of health risks inherent in same or opposite gender sexual contact.

## INTRODUCTION

### Background

According to a nationally representative 2012 Gallup survey, approximately 3.5% of U.S. adults aged 18 or over self-identify as lesbian, gay, bisexual, or transgender.^1^ Disparities in health outcomes and health behaviors exist between lesbian, gay, and bisexual (LGB) populations and heterosexual populations, including poorer mental health and less overall access to care and preventive services.^2^ LGB adults have thus been reported to seek treatment in emergency departments (EDs) at rates that are higher than the overall population.^3^,^4^ Increased ED use by LGB patients may be promoted by a variety of factors, including delayed routine healthcare due to reluctance to disclose sexual information to primary care providers.^5^–^7^ Further, mood, anxiety and substance-use disorders may be more prevalent in some LGB cohorts, both male and female.^8^,^9^ LGB patients may present to EDs seeking immediate onsite access to specialty mental health treatment, as well as expedited access to community-based mental health and chemical dependency treatment after ED discharge.^10^

Sexual orientation can be examined within three separate but related constructs: 1) self-defined sexual orientation; 2) sexual fantasy or desire; and 3) sexual behavior. This three-part definition is consistent with published recommendations,^11^–^13^ and was recently used in a population-based survey of substance abuse in U.S. adults aged 18 and over.^14^ Prior work employing this three-part approach suggests that risk of mood and anxiety disorders may be higher among people who affirmatively self-identify as LGB, but not in those who simply acknowledge experiencing same-sex attraction or behaviors without self-defining as LGB.^15^

The Institute of Medicine (IOM) issued a report on LGBT health in 2011, highlighting the gaps in health research on this population.^16^ This report, entitled “*The Health of Lesbian, Gay, Bisexual, and Transgender People: Building a Foundation for Better Understanding,”* recommended addressing several priority research areas, including first adding measures to screen patients for sexual identity in a variety of clinical settings, including the ED. However, data on sexual identity are not routinely collected in most ED settings. Reasons are varied and may include time constraints, unawareness of the heightened risk of medical and psychiatric morbidity associated with identifying as LGB, or with provider discomfort asking these particular questions.^17^ The latter barrier seems less likely in psychiatric EDs, since comprehensive biopsychosocial assessment usually involves detailed questions about a variety of other highly personal matters.

### Objective

As a component of a larger study exploring suicide risk in ED patients, we explored whether a subset of those patients would readily disclose their sexual orientation, gender attraction and prior sexual experiences in the context of research evaluation. Sexual orientation was measured along three dimensions: a self-description of sexual orientation (How would you describe your sexual orientation?: heterosexual, gay/lesbian, bisexual, not sure), a self-description of sexual attraction (Who are you attracted to sexually?: only opposite sex, mostly opposite sex, equal, mostly same sex, only same sex), and the gender of any prior sexual partners (Who have you had sex with?: only opposite sex, only same sex, both, never had sex). We tracked willingness of ED patients to answer these three questions in the context of a full suicide risk assessment. We also examined demographic and clinical characteristics of patients who self-identified as heterosexual versus those who did not. Finally, we examined whether self-identification as heterosexual or otherwise was a valid proxy for actual past sexual experiences in this sample.

## METHODS

In a study examining standardized suicide risk assessment,^18^ we enrolled participants (n=177) who presented for psychiatric treatment at three large urban emergency departments in New York City (n=68), Rochester, NY (n=55), and Philadelphia, PA (n=54). Each of these facilities had a specialty psychiatric emergency service as a component of the general ED. All subjects provided written informed consent for participation and were interviewed by trained research staff. Primary Diagnostic and Statistical Manual of Mental Disorders, 4th edition (DSM-IV) Axis I diagnoses were collapsed into groupings of mood, psychosis, substance-related disorders, or other types of disorders. We used t-tests to compare groups for continuous variables and chi-square tests to compare categorical variables.

## RESULTS

Of 177 participants, none (0%) refused to respond to the categorical question about sexual orientation and 168/177 (94.9%) also agreed to provide information about prior sexual partners. One hundred subjects (100/109; 91.7%) provided information about current sexual attraction toward either gender (attraction was not asked of Columbia subjects). Of all 177 participants, 154 (87.0%) self-identified as heterosexual, 11 (6.2%) as bisexual, 10 (5.6%) as gay or lesbian, and 2 (1.1%) indicated they were not sure. Most subjects were female (57.4%), with a mean age of 36.1 years (SD 12.4). Over half (51.1%) were white, whereas 23.6% were African-American, 19.8% were Hispanic, and 5.5% self-identified as members of another race. To conserve sample size, all subjects who did not self-identify as heterosexual were combined for further analyses. As shown in Table 1, while more LGB patients had presented to the ED for reasons of suicide attempt or self-injury (73.9%) then heterosexual patients (63.6%), this difference did not reach statistical significance. As compared with heterosexual patients, LGB patients were significantly younger and more likely to be non-white, but did not differ significantly in terms of education, income, employment, or religious affiliation or participation. Females appeared more likely to identify as non-heterosexual, although this did not reach statistical significance. Over half (55.8%) of heterosexual patients were parents while approximately one quarter (26.1%) of LGB patients were parents, a statistically significant difference.

Table 2 displays clinical characteristics of the two groups. LGB participants did not differ from their heterosexual counterparts in terms of lifetime suicide attempt rate or lifetime history of any mood, substance-related, psychotic spectrum, or other DSM-IV Axis I disorder. Current Global Assessment of Function score averages were in the 30s range for both groups, indicating significant impairment due to a mental disorder. Likewise while 10% more LGB subjects met diagnostic criteria for borderline personality disorder when compared with the heterosexual group, these differences were not statistically significant.

Data on self-described sexual attraction and previous sexual partners revealed that 5.6% (5/89) of participants that self-categorized their sexual orientation as ‘heterosexual’ reported sexual attraction as other than ‘only opposite sex,’ and 10.3% (15/142) of sexually active ‘heterosexual’ participants reported previous same-gender sexual partners. Conversely, all 11 participants who defined their sexuality as other than heterosexual reported sexual attraction as other than ‘only opposite sex,’ and a majority (63.6%, 14/22) of sexually active non-heterosexual participants nonetheless reported prior opposite-gender sexual partners.

## DISCUSSION

No ED subjects refused to provide a self-definition of sexual orientation. Almost all (94.9%) were also willing to provide information about the gender of prior sexual partners in the context of a health examination. A majority (91.7%) also agreed to answer a question about current sexual attraction toward either or both genders. This supports the practicality of a three-question screening strategy in the ED environment, where privacy is a major concern.

LGB people were represented in this sample of ED subjects at rates similar or higher than their reported proportions in the overall U.S. populace. The LGB subjects identified were younger, perhaps reflecting a generational shift in willingness to disclose non-heterosexual orientations. LGB subjects were also more likely to be racially diverse, but did not vary from heterosexuals in terms of the other socioeconomic or clinical indices examined.

Finally, there was incomplete concordance between self-defined sexual orientation and actual sexual experience, with about one in 10 self-attributed heterosexuals disclosing previous same-sex encounters, and six in 10 self-attributed non-heterosexuals describing previous opposite-sex encounters. If one of the main purposes of posing these questions is to ascertain health risks, then simply asking the patient to define their sexual identity will likely be misleading in some instances.

## LIMITATIONS

This study has important limitations, including a small sample size. We did not ask about the timing, duration, extent, potential medical or psychological consequences or personal meaning of the sexual activities identified. We also did not ask specifically about transgendered people. We did not explore reasons for subject reluctance to answer any of the questions about sexual identity. Questions were asked by research staff and not by ED clinicians. Questions were also asked in psychiatric EDs and not medical EDs, so findings may not generalize.

## CONCLUSION

Assessing patients’ sexual orientation in the ED by a three-question approach appeared feasible in the ED and acceptable to ED patients. However, since many patients have sexual experiences not suggested by simple labels, self-report of sexual identity alone may not inform clinicians of health risks inherent in same or opposite gender sexual contact.

## Figures and Tables

**Table 1:** Demographic characteristics of participants in study of self-defined sexual orientation.

	Self-defined sexual orientation		Test b	
	
Demographic characteristics a	Heterosexual n=154 (%)	Other than heterosexual n=23 (%)	Statistic	p-value
Study group				
Attempter or self-injurer	98 (63.6)	17 (73.9)	0.929	0.335
Non-attempter/non-self-injurer	56 (36.4)	6 (26.1)		
Gender				
Male	70 (45.5)	6 (26.1)	3.064	0.080
Female	84 (54.5)	17 (73.9)		
Age: years, mean (SD)	36.5 (12.1)	30.7 (12.9)	2.154	0.033
Race				
White	81 (52.6)	7 (30.4)	3.932	0.047
Non-white	73 (47.4)	16 (69.6)		
Religious affiliation				
Catholic/Protestant/Jewish	91 (60.3)	11 (47.8)	1.273	0.259
Other	60 (39.7)	12 (52.2)		
Frequence of religious services attendance				
Once or more per month	55 (36.2)	4 (17.4)	3.157	0.076
Less than once per month	97 (63.8)	19 (82.6)		
Employed or student c - Yes	50 (33.1)	8 (34.8)	0.025	0.874
Household income				
Less than 20K	83 (61.9)	13 (68.4)	0.299	0.585
20K and greater	51 (38.1)	6 (31.6)		
Highest education				
Less than high school graduate	43 (27.9)	3 (13.0)	2.303	0.129
High school grad and higher	111 (72.1)	20 (87.0)		
Number of children: mean (SD)	1.23 (1.54)	0.74 (1.51)	1.442	0.151
Parent (at least 1 child) - Yes	86 (55.8)	6 (26.1)	7.099	0.008

a Totals do not always equal 237 due to missing data.

b Test comparing ‘straight’ and ‘other than straight’ groups. Continuous variables tests are t-tests, categorical variables tests are Chi-squares.

c ‘Yes’ includes full-time or part-time, ‘no’ includes unemployed, retired, homemaker, disabled, other.

**Table 2:** Clinical characteristics for heterosexual and other than heterosexual groups.*

Continuous variables [mean (SD)]	Heterosexual (n=154)	Other (n=23)
Number of lifetime suicide attempts	2.03 (2.59)	1.96 (2.10)
Global assessment of function (GAF)	35.39 (9.98)	31.83 (10.51)
Categorical variables [n (%)]		
Mood disorder		
Yes	124 (84.9)	20 (95.2)
No	22 (15.1)	1 (4.8)
Substance disorder		
Yes	67 (45.9)	9 (42.9)
No	79 (54.1)	12 (57.1)
Anxiety disorder		
Yes	15 (10.3)	2 (9.5)
No	131 (89.7)	19 (90.5)
Psychotic disorder		
Yes	12 (8.2)	0 (0)
No	134 (91.8)	21 (100)
Other axis-I disorder		
Yes	5 (3.4)	1 (4.8)
No	141 (96.6)	20 (95.2)
BPD (meets dx criteria)**		
Yes	32 (34.0)	7 (43.8)
No	62 (66.0)	9 (56.3)

*BPD,* borderline personality disorder

* Totals do not always equal 177 due to missing data.

** Borderline Personality Disorder (BPD) information not obtained for Rochester sample.
